# Case Report: Locally advanced clear cell renal cell carcinoma with pathological complete response following nephrectomy and thrombectomy after nivolumab and cabozantinib treatment

**DOI:** 10.3389/fonc.2025.1537973

**Published:** 2025-09-25

**Authors:** Hayato Yoshioka, Hiroyuki Kitano, Kohei Kobatake, Yohei Sekino, Akira Ishikawa, Keisuke Goto, Akihiro Goriki, Keisuke Hieda, Kouji Arihiro, Nobuyuki Hinata

**Affiliations:** ^1^ Department of Urology, Graduate School of Biomedical and Health Sciences, Hiroshima University, Hiroshima, Japan; ^2^ Department of Molecular Pathology, Graduate School of Biomedical and Health Sciences, Hiroshima University, Hiroshima, Japan; ^3^ Department of Pathology, Graduate School of Biomedical and Health Sciences, Hiroshima University, Hiroshima, Japan

**Keywords:** deferred cytoreductive nephrectomy, ccRCC, immune checkpoint inhibitor, VEGFR-TKI, pathological complete response, robot-assisted nephrectomy, nivolumab, cabozantinib

## Abstract

The recent introduction of systemic anticancer therapies (SACT) and immune checkpoint inhibitors in cancer management has led to reports on the usefulness of deferred cytoreductive nephrectomy (dCN) following vascular endothelial growth factor receptor-tyrosine kinase inhibitor and immune checkpoint inhibitor combination therapy (VEGFR-TKI + ICI) for metastatic renal cell carcinoma (RCC), as well as nephrectomy after VEGFR-TKI + ICI combination therapy for initially unresectable locally advanced RCC. However, the optimal approach to SACT and the suitable patient profiles for these approaches remain unclear. We report the case of a 73-year-old man with stage III RCC accompanied by venous invasion, initially diagnosed as unresectable. Following VEGFR-TKI + ICI combination therapy with nivolumab and cabozantinib, he underwent nephrectomy and thrombectomy, resulting in a pathological complete response (pCR). The patient was diagnosed with left RCC after a tumor measuring 80 × 60 mm with tumor thrombus in the left renal vein was confirmed (cT3aN0M0), and subsequent percutaneous biopsy performed prior to embolization revealed clear cell histology. The tumor size was reduced following treatment with nivolumab and cabozantinib. Robot-assisted left nephrectomy was subsequently performed. Postoperative pathology tests confirmed no malignant findings, suggesting pCR. Conventionally, cytoreductive nephrectomy is performed prior to SACT; however, there has been an increase in dCN use. In this case, the combination of nivolumab and cabozantinib led to the pCR of unresectable RCC, suggesting that VEGFR-TKI + ICI combination therapy may exert a strong tumor-reducing effect and could contribute to the establishment of an optimal SACT regimen prior to dCN or nephrectomy in patients with locally advanced RCC.

## 1 Introduction

The CARMENA study demonstrated that compared to systemic anticancer therapy (SACT; sunitinib) alone, immediate cytoreductive nephrectomy (iCN) performed prior to SACT did not improve overall survival in patients with intermediate- or poor-risk clear cell renal cell carcinoma (ccRCC) (1). However, with the advent of vascular endothelial growth factor receptor-tyrosine kinase inhibitors (VEGFR-TKIs) and immune checkpoint inhibitors (ICIs), the effectiveness of deferred cytoreductive nephrectomy (dCN) following SACT has been reported in patients with metastatic ccRCC (2, 3). Nonetheless, the optimal SACT strategy and the specific patient populations that may benefit from dCN remain unclear.

Herein, we report a case of locally advanced ccRCC treated with a combination of nivolumab and cabozantinib followed by robot-assisted nephrectomy and thrombectomy, which resulted in a pathological complete response (pCR).

## 2 Case presentation

A 73-year-old man presented with a chief complaint of gross hematuria. His past medical history was unremarkable, and there was no family history of ccRCC. In 202X, he was referred to our department following evaluation by a previous physician. An iodine contrast-enhanced computed tomography (CT) scan revealed an 80 × 60 mm renal tumor with a tumor thrombus extending into the left renal vein (Mayo Clinic Level 0). Iodine contrast-enhanced whole-body CT revealed no apparent signs of distant metastasis.

A percutaneous biopsy of the renal mass confirmed the diagnosis of ccRCC in the left kidney, and the disease was clinically staged as cT3aN0M0 (stage III) according to the Tumor Node Metastasis classification. The patient’s height was 172.5 cm, and his body weight was 67.0 kg. Laboratory results relevant to the IMDC criteria were as follows: hemoglobin, 14.4 g/dL; corrected calcium, 9.0 mg/dL; neutrophil count, 4010/μL; and platelet count, 225,000/μL.

Due to the presence of venous invasion, the tumor was initially considered unresectable, and SACT was initiated. From Y + 1, 202X, the patient received combination therapy with nivolumab (480 mg intravenously every 4 weeks) and cabozantinib (40 mg orally once daily).

**Figure 1:**
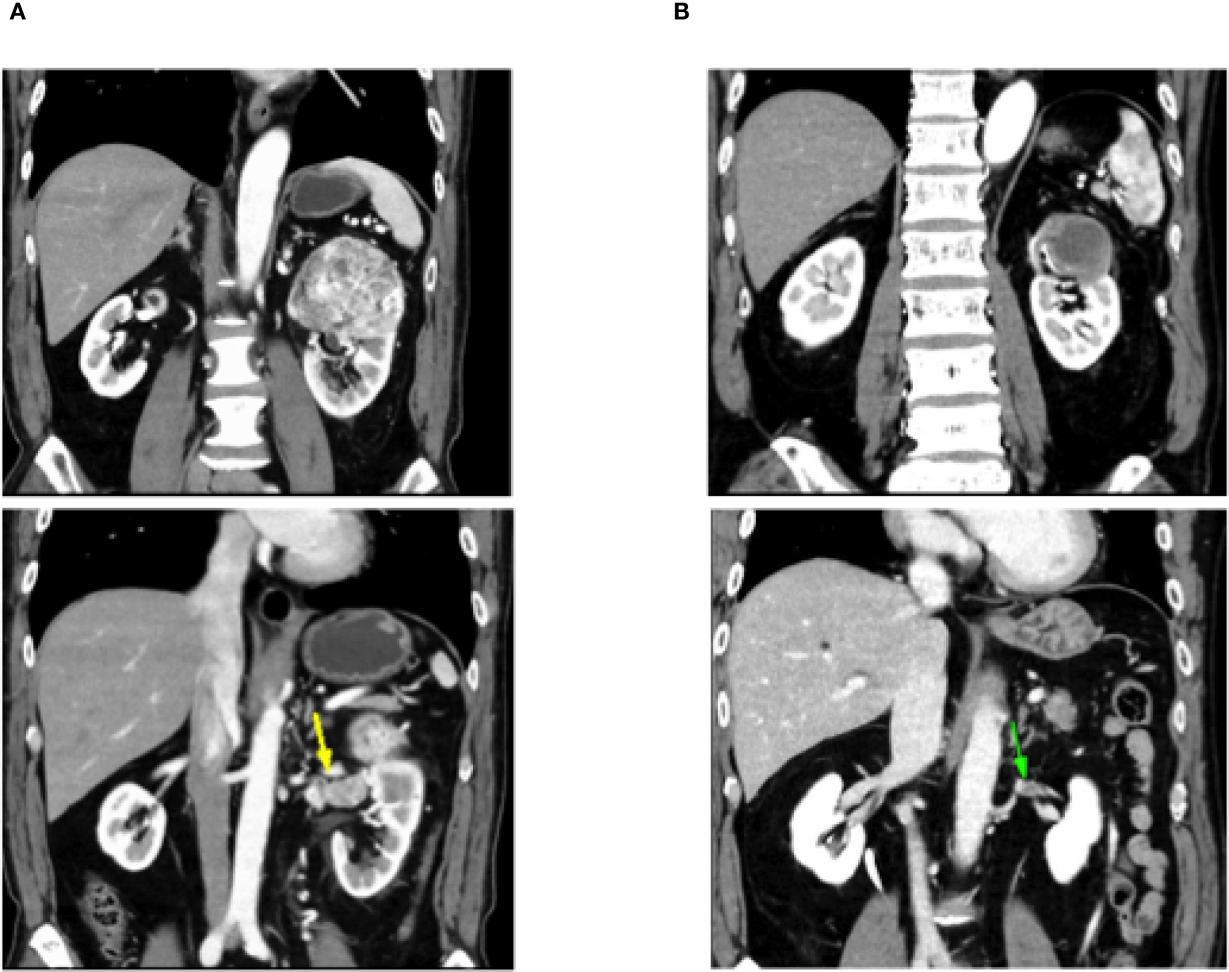
Follow-up computed tomography scan shows that the left renal tumor shrank from 80 × 60 mm to 47 × 35 mm and the tumor thrombus is markedly reduced. **(A)** CT before Nivolumab + cabozantinib. **(B)** CT after Nivolumab + cabozantinib.

During treatment, he developed a grade 1 skin rash and grade 1 hoarseness, both of which were resolved with conservative management. Two months later, he developed grade 2 hypothyroidism, which was diagnosed as painless thyroiditis based on endocrine evaluation, including measurements of thyroid function (FT3, FT4), thyroid-stimulating hormone, and thyroid autoantibodies (anti-TPO, anti-thyroglobulin, and TRAb). Levothyroxine replacement therapy was initiated and titrated to maintain euthyroidism. A follow-up CT scan after five courses of SACT showed that the renal tumor had decreased in size from 80 × 60 mm to 47 × 35 mm, and the tumor thrombus had markedly regressed (**Figure 1**). The tumor was therefore considered resectable, and the patient underwent robot-assisted left nephrectomy and venous thrombectomy.

The operation lasted 2 hours and 30 minutes, with an insufflation time of 1 hour and 55 minutes and a console time of 1 hour and 35 minutes. The intraoperative blood loss was minimal (5 mL), and the resected specimen weighed 616 g. Intraoperative findings revealed no residual tumor thrombus in the renal vein.

**Figure 2:**
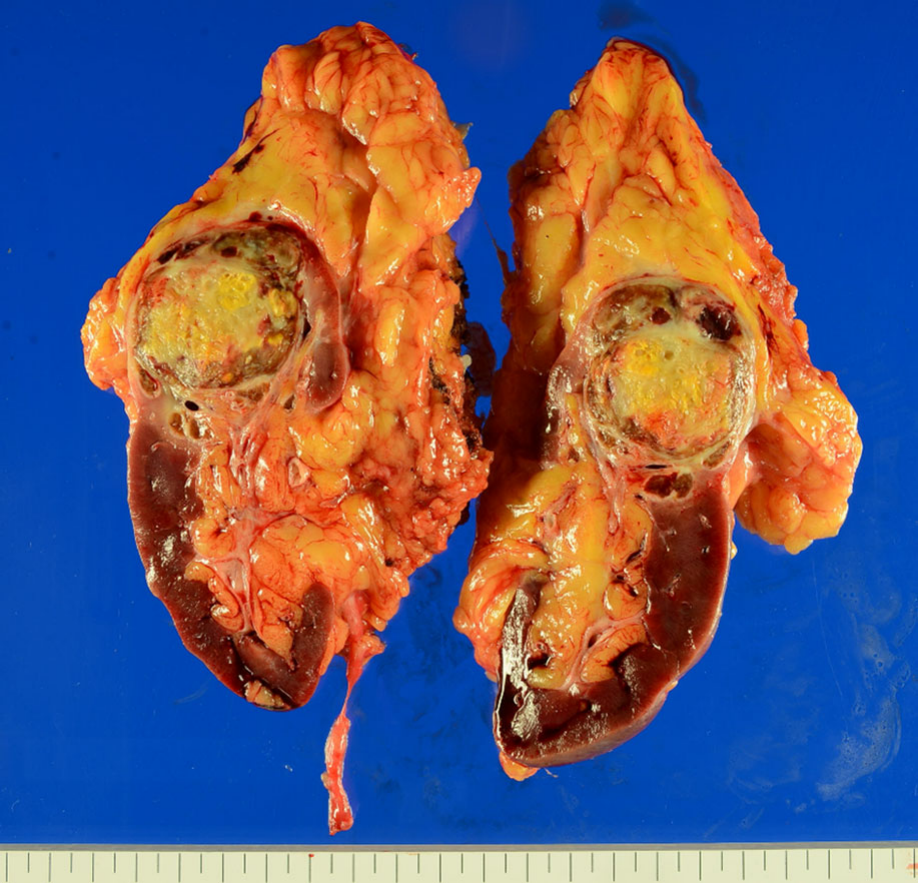
Left nephrectomy specimen (size: 16 × 15 × 2 cm). Macroscopically, a brown-yellow tumor with a maximum diameter of 4.5 cm was observed.

**Figure 3:**
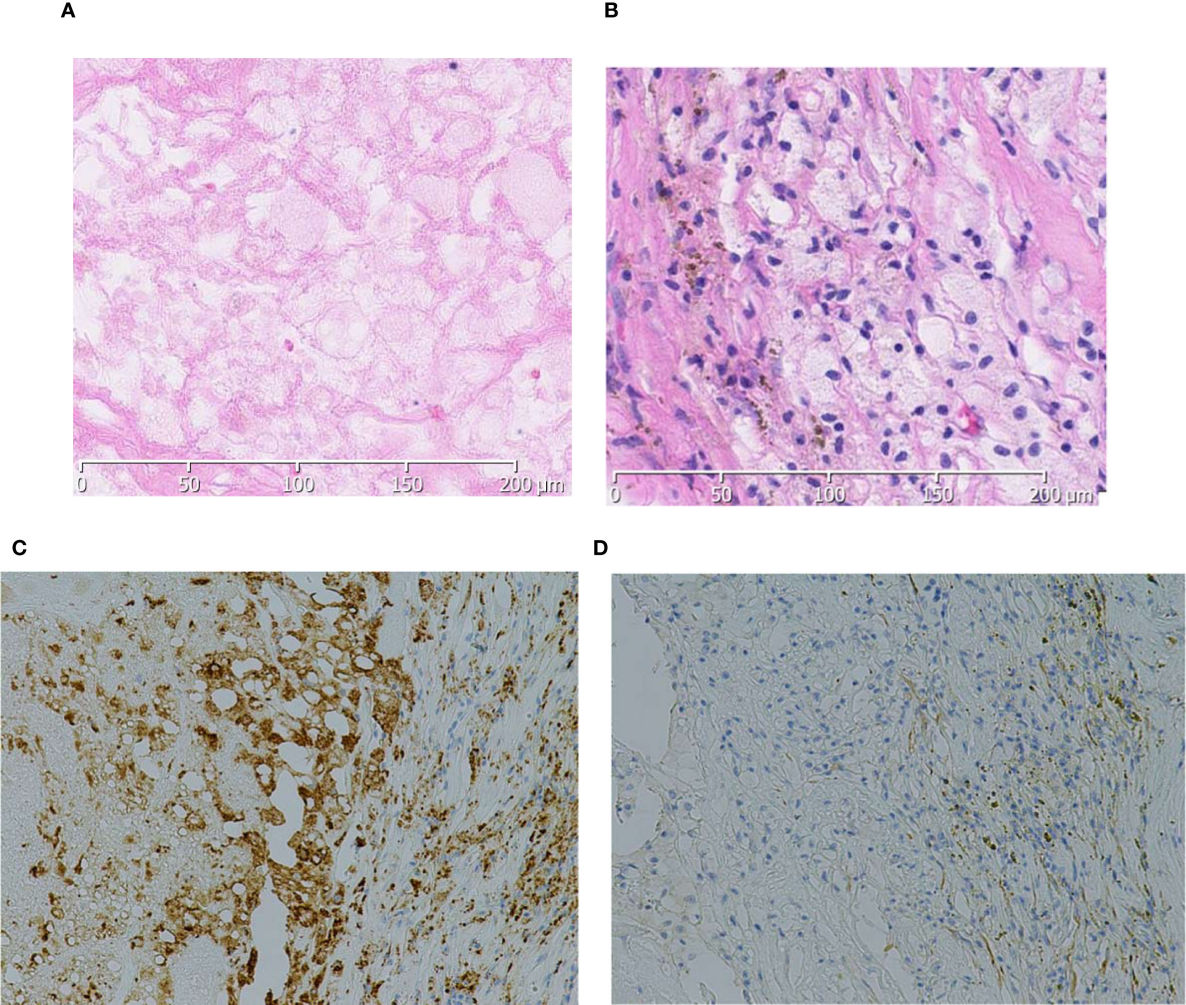
Histologically, extensive necrosis is observed within the tumor area **(A)**. In the peripheral regions, inflammatory granulation tissue is noted **(B)**, and the cells are positive for CD68 **(C)** and negative for CKAE1/AE3 **(D)**.

The postoperative course was uneventful; no postoperative complications of Clavien–Dindo grade I or higher were observed, and the patient was discharged on postoperative day 8. Gross pathological examination revealed a yellow–brown solid tumor measuring 4.5 cm in greatest diameter within a fibromuscular capsule (**Figure 2**). No residual tumor was observed in the renal vein on pathological examination. Histologically, extensive coagulative necrosis was observed throughout the tumor, and inflammatory granulation tissue was seen in peripheral areas. To confirm the absence of residual viable tumor cells, immunohistochemical staining was performed. The cells were positive for CD68 and negative for CKAE1/AE3, indicating macrophages but no residual carcinoma cells. Based on these findings, the tumor was considered to have achieved a pCR following combination therapy with nivolumab and cabozantinib (**Figure 3**).

**Figure 4:**
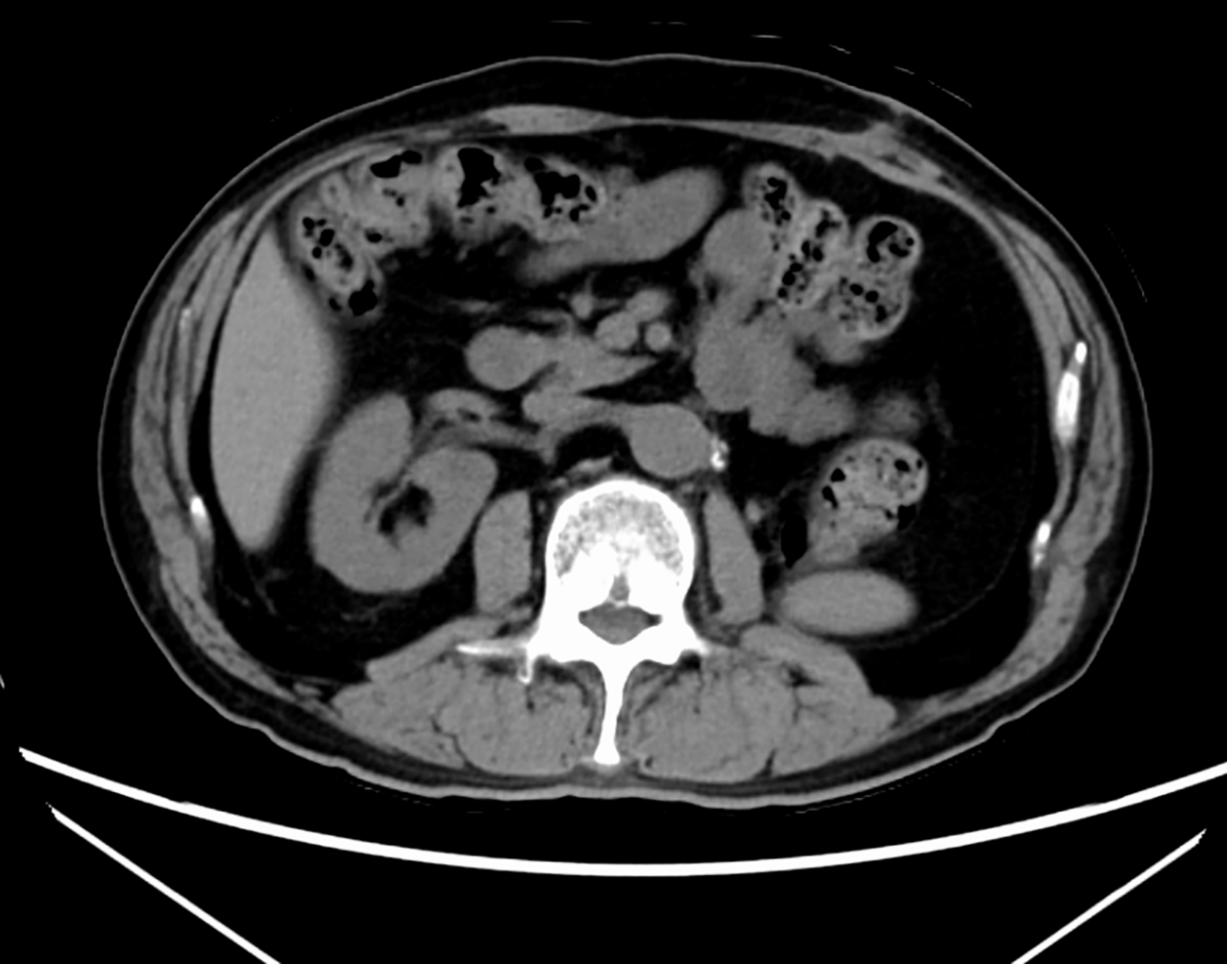
No evidence of recurrence or metastasis is seen at the 18-month follow-up.

No adjuvant SACT was administered. The patient has since been followed with regular CT imaging, and at 18 months postoperatively, no evidence of recurrence or metastasis has been observed (**Figure 4**).

## 3 Discussion

We report a case of advanced ccRCC with a tumor thrombus in the renal vein, classified as stage III, that was initially deemed unresectable. After SACT with nivolumab and cabozantinib, significant tumor shrinkage was achieved. The patient subsequently underwent robot-assisted nephrectomy and venous tumor thrombectomy, and pathological examination confirmed a pCR.

To our knowledge, this is the first reported case of locally advanced ccRCC achieving a pCR with VEGFR-TKI + ICI therapy following nephrectomy after pretreatment with nivolumab and cabozantinib.

Traditionally, cytoreductive nephrectomy (CN) has been performed prior to SACT; however, with the advent of ICI combinations and ICI + VEGFR-TKI regimens, there has been a growing number of reports on nephrectomy—including dCN—being performed after SACT for patients with metastatic ccRCC, owing to the significant tumor-reducing effects of these agents (4). The SURTIME trial—a randomized phase III trial designed to compare the efficacy of iCN versus dCN in patients with metastatic ccRCC—evaluated the efficacy of dCN preceded by sunitinib. Although the trial did not reach its target patient enrollment number, a trend toward longer median overall survival was observed in the dCN group compared to the iCN group (5).

The options for SACT in metastatic, advanced ccRCC have expanded, with combination therapies centered on ICI–based regimens gaining attention in recent years. ICI combination therapies can be broadly categorized into two main types: ICI + ICI and ICI + VEGFR-TKI. ICI + ICI includes nivolumab + ipilimumab, while ICI + VEGFR-TKI includes pembrolizumab + axitinib, nivolumab + cabozantinib, pembrolizumab + lenvatinib, and avelumab + axitinib. All of these therapies are approved in Japan as first-line treatments for unresectable advanced RCC and metastatic RCC (5–9).

Appropriate SACT for patients scheduled to undergo nephrectomy following SACT, including dCN, has yet to be established. The NORDIC-SUN trial evaluated the efficacy of dCN in patients with synchronous metastatic ccRCC, classified as intermediate or poor risk based on the IMDC risk classification, in combination with ICI-based regimens (10). The IMDC criteria are widely used for prognostic stratification and predictive assessment in metastatic RCC. Although originally developed for metastatic disease, modified IMDC models have been explored in localized and locally advanced RCC, as reported by Horie et al. (11). In the present case of locally advanced RCC, we present the laboratory parameters according to the IMDC classification, acknowledging these prior attempts at validation outside the metastatic setting. The PROBE trial is currently assessing the primary endpoint of overall survival in patients with metastatic ccRCC undergoing dCN following ICI combination therapy, with final reports pending (12).

**Figure 5:**
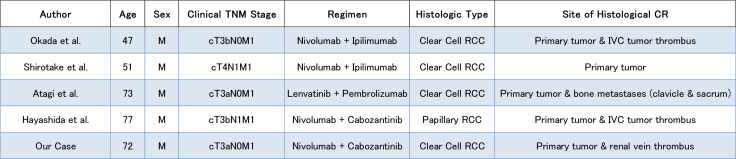
Summary of previously reported cases of renal cell carcinoma with a pathological complete response following immune checkpoint inhibitor-based therapy, including the present case.

The present patient had locally advanced ccRCC (cT3aN0M0) with venous invasion. After SACT with nivolumab + cabozantinib, the patient underwent nephrectomy and thrombectomy, which demonstrated a pCR. Previous reports of pCR have included cases in which nephrectomy was performed following combination therapy with ICIs (4, 13–15) (**Figure 5**). Okada et al. (13) reported the pCR of both the primary renal tumor and inferior vena cava tumor thrombus in a patient with intermediate-risk metastatic ccRCC (cT3bN0M1) following dCN after treatment with nivolumab + ipilimumab (ICI + ICI). Similarly, in 2022, Shirotake et al. (14) reported the case of a 51-year-old man with metastatic ccRCC (cT4N1M1) having a pCR in the primary renal tumor after dCN with nivolumab + ipilimumab therapy. Furthermore, Atagi et al. (4) reported the case of a 73-year-old man with intermediate-risk metastatic ccRCC (cT3aN0M1) who underwent dCN and metastasectomy of the clavicle and sacrum after treatment with pembrolizumab and lenvatinib, and achieved a pCR in both the primary tumor and bone metastases. Regarding nivolumab and cabozantinib, Hayashida et al. (15) reported the case of a 77-year-old man with papillary RCC (cT3bN1M1) who achieved a pCR of both the primary tumor and the tumor thrombus following dCN after treatment with nivolumab and cabozantinib.

As mentioned above, a pCR has been observed in cases where ICI + ICI or ICI + VEGFR-TKI was used as pretreatment for nephrectomy. In our case, treatment with nivolumab and cabozantinib resulted in a pCR of advanced ccRCC. Regarding the choice of drug treatment for dCN, nivolumab + ipilimumab has been reported by Motzer et al. (16) over a follow-up period of >5 years, indicating that long-term remission can be expected in some patient. However, subgroup analyses have raised concerns regarding potential tumor growth in the primary renal site, as opposed to the treatment effects observed on metastases (16, 17). In contrast, the objective response rate has been reported to exceed 50% for ICI + VEGFR-TKI regimens, such as nivolumab + cabozantinib and pembrolizumab + lenvatinib, with particularly low disease progression rates (5–6%) after treatment initiation (6–9). In addition, a relatively high tumor reduction effect is anticipated for primary kidney tumors (18).

Both ICI + ICI and ICI + VEGFR-TKI therapies require monitoring of immune-related adverse events (irAEs). Furthermore, the high risk of severe irAEs with ICI + ICI therapy remains a concern. Such irAEs can delay the initiation of surgical treatment and often require high-dose systemic corticosteroids; consequently, they may significantly and adversely affect oncological outcomes and quality of life. Moreover, VEGFR-TKIs inhibit angiogenesis and reduce tumor vascularity, which may contribute to decreased intraoperative blood loss during nephrectomy, supporting the use of ICI + VEGFR-TKI as a suitable SACT when nephrectomy is planned following SACT, including dCN (19).

The 5-year survival rate for stage III ccRCC has been reported to range from approximately 53% to 75% (20). In the present case, a pCR was achieved following nephrectomy after SACT, suggesting the potential for favorable long-term outcomes.

A notable limitation of this case is that although tumor disappearance was observed, long-term follow-up was not conducted; therefore, ongoing observation is necessary to monitor for potential recurrence.

In conclusion, the present case involved advanced ccRCC with a venous tumor thrombus, in which robot-assisted nephrectomy and venous thrombectomy were performed following pretreatment with the ICI + VEGFR-TKI combination therapy of nivolumab and cabozantinib, resulting in a pCR. Further accumulation of similar cases is warranted to clarify the efficacy of this multimodal anticancer treatment approach that combines systemic therapy with the operative procedure.

## 4 Patient perspective

The patient expressed relief and satisfaction after completing the treatment and surgery. He was especially encouraged by the absence of recurrence and metastasis at the 18-month follow-up. Although he had some mild adverse events during systemic therapy, such as skin rash, hoarseness, and hypothyroidism, these were manageable with conservative treatment. He remains under regular clinical follow-up and is hopeful about maintaining a good long-term prognosis.

## Data Availability

The original contributions presented in the study are included in the article/supplementary material. Further inquiries can be directed to the corresponding author.
